# Meat inspection of pigs slaughtered in Norwegian abattoirs: insights from variance partitioning analysis

**DOI:** 10.1186/s12917-026-05401-2

**Published:** 2026-03-11

**Authors:** Kristine Paulsen Eggen, Hilde Vinje, Camilla Kielland, Marit Nesje, Ingeborg Sveinsdottir, Ingrid Toftaker

**Affiliations:** 1 Department of Production Animal Clinical Sciences, Faculty of Veterinary Medicine, Norwegian University of Life Sciences, Ås, Norway; 2 Division Land Animals and Abattoir, The Norwegian Food Safety Authority, Brumunddal, Norway; 3 Faculty of Chemistry, Biotechnology and Food Science, Norwegian University of Life Sciences, Ås, Norway; 4 Section Terrestrial Animal Health and Welfare, The Norwegian Veterinary Institute, Ås, Norway

**Keywords:** meat inspection, variance partitioning analysis, pig production, surveillance, animal welfare

## Abstract

**Background:**

Routinely recorded meat inspection data represent a valuable source of information for animal health and welfare surveillance. However, their utility for secondary purposes depends on data quality and the consistency of recorded findings. This study aims to describe the data recorded during the slaughter process for Norwegian pigs, the feasibility of combining datasets, and estimate the contribution of farm, abattoir, and external factors to the overall variation in recorded findings, using meat inspection records from October 2021 to March 2024 from 15 abattoirs, comprising 75 828 batches.

**Results:**

Meat inspection data are recorded at three stages of the pig slaughter process, resulting in three separate databases. Merging of these datasets was not possible at the individual pig level with adequate completeness, and substantial manual data processing was required to combine data at the batch level. Post-mortem inspection is conducted at two stages: routine inspection of all carcasses, recorded as “extended disease registrations” (EDR) and assessment of carcasses suspected of requiring condemnation, documented as “post-mortem findings”. The variation in the most frequent EDR findings and post-mortem findings was estimated using variance partitioning. EDR findings showed low variation between abattoirs, particularly for “pericarditis and/or pleuritis” and “open tail wounds”, suggesting consistent detection between abattoirs. In contrast, the post-mortem findings «systemic disease», “abscesses/phlegmons” and “gastrointestinal disease” showed substantial abattoir-level variance, indicating inconsistencies in recording practices. Season had minimal effect on the variance components, while day of week only affected the post-mortem finding «gastrointestinal disease».

**Conclusion:**

EDR findings were consistently detected across abattoirs, suggesting their potential as a reliable source for animal health and welfare surveillance. In contrast, substantial variation in certain post-mortem findings highlights the need for harmonization and standardization of recording practices. Future improvements in data quality and traceability are essential for robust secondary use of meat inspection data in surveillance.

**Supplementary Information:**

The online version contains supplementary material available at 10.1186/s12917-026-05401-2.

## Introduction

In the European Union, all animals raised for meat production must undergo ante-mortem (AM) and post-mortem (PM) inspection at slaughter, as mandated by EU Regulation 2017/625 [1] and further detailed in Regulation 2019/627 [2]. Upon arrival at the abattoir, live pigs undergo AM inspection, which includes reviewing of food chain information and observing the animals during unloading or in lairage. The purpose is to detect signs of compromised animal health and welfare, notifiable diseases, or other conditions that may affect food safety [2]. Animals that pass the AM inspection are approved for slaughter.

Following slaughter, carcass and viscera undergo PM inspection to detect and remove macroscopic abnormalities that may compromise suitability for human consumption [2, 3]. This process primarily involves visual inspection; however, if suspicious findings are observed, further procedures such as palpation, incisions, or laboratory testing may be performed [2]. While the main purpose of meat inspection is to ensure food safety, it also provides valuable information on animal health and welfare, and the zoo-sanitary status of farms. Thus, meat inspection is essential in broader surveillance systems for pig health and welfare [3–5].

The interest in using routinely recorded data for animal health and welfare surveillance has increased in recent years [6]. Several studies highlight the potential of meat inspection data for this purpose, as well as for providing feedback to producers to improve herd management [7–11]. However, systematic collection and use of these data remain underutilized in most European countries [3]. To fully realize their value, it is essential that the data are accessible, reliable, and effectively analysed to support decision-making processes for various stakeholders, from farmers seeking feedback on herd health and management to veterinary authorities conducting risk-based inspections in farms [9]. Reliable secondary use of meat inspection data also requires a thorough understanding of data quality, including consistency, completeness, and validity [12]. Considerable differences in coding systems and meat inspection practices across European countries complicate data comparability between nations [13]. Several European studies have identified challenges to data quality that must be addressed [7, 9, 11, 14–18]. One challenge is the low sensitivity for certain lesions and types of contamination during routine meat inspection. A Danish study using latent class analysis found that inspectors had high specificity (Sp) but low sensitivity (Se), particularly for detecting parasitic and intestinal disease, while they found that veterinary researchers had high Se but slightly lower Sp [17]. The authors suggested inspectors may require more “typical” clinical signs to classify a pig as positive, potentially leading to reduced data quality and underestimation of disease prevalence. Other European studies report moderate to high variability in post-mortem findings, both between abattoirs and between inspectors within the same abattoir [11, 14–16, 18], suggesting that factors such as local routines, training and interpretation might influence consistency. The extent of variation between abattoirs or inspectors depend on the type of meat inspection finding [14–16]; moreover, which findings are recorded most consistently also varies between studies and the lesion code system in place [14, 16].

To better understand sources of variation in meat inspection data, several studies have applied variance partitioning methods [14–16]. This statistical approach quantifies how much of the observed variation can be attributed to different grouping factors (clusters), such as farm, abattoir, or inspector. The aim of this approach is to disentangle true biological variation from variation introduced by the data collection process, such as differences in recording practices between abattoirs. Recognizing that disease prevalence may also be affected by external factors, Schleicher et al. [16] found that accounting for external factors such as season, farm type and piglet producers, reduced farm-level variance but left inspector-level variance largely unchanged. These results highlight the importance of accounting for external factors to accurately attribute variance to farms and abattoirs.

In Norway, approximately 1.5 million pigs are slaughtered annually from about 2 200 farms [19, 20]. Official meat inspection is carried out by official veterinarians and auxiliaries employed by the Norwegian Food Safety Authority (NFSA), in accordance with implemented EU regulations [21, 22]. Some inspection findings, notably “open tail wounds”, “abscesses and infected wounds”, and “arthritis”, are used by the livestock industry for producer feedback and advisory purposes [23, 24], much of the data remains underutilized. While a study has briefly compared meat inspection processes and findings across seven European countries [13], a comprehensive description of Norwegian data collection, the feasibility of dataset linkage, and data quality is currently lacking.

The NFSA is responsible for enforcing the animal welfare legislation, conducting risk-based farm inspections and follow-up to ensure compliance. Although meat inspection data may inform these assessments, there is currently no standardized approach for integrating abattoir data. According to EFSA, main limitations to the value of meat inspection data is the lack of systematic data capture, analysis and communication between abattoirs and countries [3]. Here, data capture refers to the consistent and standardized recording of relevant findings during meat inspection, including identification, classification and documentation for each animal or batch. Improved knowledge of data quality is a crucial first step towards enhancing its utility, and can subsequently support more targeted regulatory inspections, better resource allocation, and ultimately improved animal health and welfare outcomes. Assessing data quality may also identify opportunities for improved calibration and standardization of meat inspection practices, supporting more reliable, data-driven use of these records.

As an initial step towards expanding the use of abattoir records for purposes such as the ones mentioned above, the objectives of this study were to: (1) describe the data collected for pigs in Norwegian abattoirs, (2) assess the feasibility of combining datasets from the meat inspection process, (3) estimate the relative contributions of farm compared to abattoir to the total variance in recorded findings, and (4) assess the effect of season on the variance component estimates, and evaluate whether day of week was associated with differences in recorded findings.

## Materials and methods

**Fig. 1:**
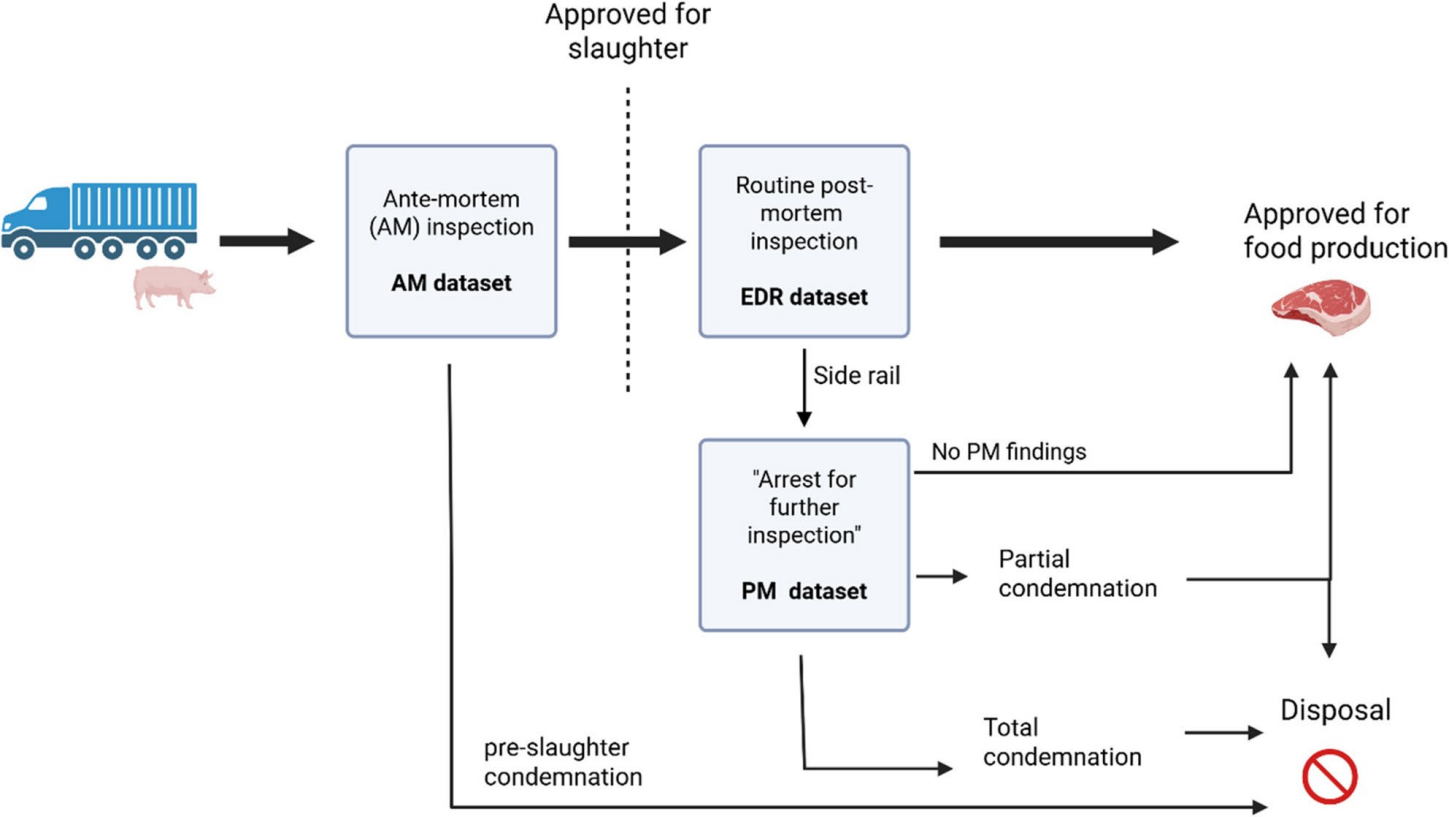
Meat inspection process and data sources for pigs in Norwegian abattoirs. Bold arrows in the figure illustrate the main slaughter line. Created with BioRender.com

This repeated cross-sectional study utilized meat inspection data from pigs slaughtered in Norwegian abattoirs. The data were provided by the NFSA and extracted from three separate databases, resulting in three distinct datasets: (1) AM records of live animal assessments, hereafter termed the *AM dataset*, (2) extended disease registrations (EDR, abbreviated USR in Norwegian) of all slaughtered pigs, referred to as the *EDR dataset*, (3) PM records of pathological findings leading to condemnation, termed the *PM dataset*. The data sources are described in detail in “Meat inspection data” section, and the data collection process is visualised in Fig. 1.

In this study, a batch of pigs is defined as animals from the same farm, slaughtered at the same abattoir on the same date. The study’s inclusion criteria were batches of pigs slaughtered between 13 October 2021 and 27 March 2024, only batches delivered to abattoirs using the EDR system and only from abattoirs with more than 100 batches slaughtered during the study period. As the EDR dataset included records of all slaughtered pigs, regardless of any disease detection, this dataset was used to define the census population in this study.

### Meat inspection data

All meat inspection findings in Norway are recorded using a standardized coding system described in the NFSA’s meat inspection instructions [25, 26]. In 2025, 17 codes for AM findings were available for pigs, eight of which are associated with conditions that may lead to pre-slaughter condemnation of individual pigs. Of these, 16 were present in the dataset, in addition to one code that has since been inactivated due to updates to the coding system. This inactivated code was included in both the dataset and analysis. AM findings are generally recorded at the batch level, and multiple findings can be recorded for each batch. If a pig is condemned pre-slaughter, the main reason for condemnation is recorded. The AM findings are recorded in NFSA’s meat inspection case file system (abbreviated MAKKS in Norwegian).

PM inspection findings in Norway are recorded using two parallel coding systems: one for EDR findings and one for PM findings. These systems serve different purposes in the meat inspection process, with EDR findings providing information relevant for herd health and welfare, and PM findings documenting reasons for condemnations. Throughout this article, we refer to findings recorded in these systems as *EDR findings* and *PM findings*, respectively. EDR findings are documented during routine PM inspection of all carcasses and include diagnostic codes of clinical importance. These codes are designed to capture findings that may be relevant for preventive herd health management and as indicators of animal health and welfare [27]. The coding system was developed collaboratively by the NFSA, the abattoirs and the industry organization, Animalia, in the 1970s, and has been in force since then [13]. Currently, EDR findings are recorded by the NFSA’s meat inspectors at most abattoirs in Norway, except for some smaller abattoirs [24]. The EDR coding system for pigs consists of eight diagnostic codes [25], which are listed and described in Table 1. Multiple findings can be recorded for each carcass, and all eight codes were represented in the EDR dataset.

**Table 1:** Description of The Extended Disease Registration (EDR) findings for pigs in Norwegian abattoirs

EDR Finding	Description
Abscesses and infected wounds	All removed abscesses and infected wounds, including well-defined cavities filled with pus and disseminated lung abscesses.
Arthritis and joint lesions	Enlarged joints and/or lymph node(s), often with excess joint fluid, thickened joint capsule, and fibrin deposits.
Pericarditis and/or pleuritis	Lesions on pleura, lungs, and/or pericardium larger than ~ 8–10 cm (relative to the size of the animal), including both acute and chronic changes.
Pneumonia	Inflammatory lung lesions > 2.5 cm, such as areas of consolidation, redness, and pus.
Ascariasis	More than five visible nodules in the liver.
Shoulder ulcers in sows	Ulcers with damaged skin, swelling and haemorrhages, recorded if grade 3 or 4 (i.e., extending into subcutaneous tissue).
Short tail/healed tail wound	Short tails ≤ 15 cm with intact skin.
Open tail wound	All open tail wounds and necrotic tails, including tail abscesses.

If a carcass shows findings raising concern about its suitability for human consumption, it is removed from the main slaughter line to a side rail, referred to as being placed “under arrest for further inspection”. At this stage, additional examinations are conducted, and PM findings are recorded using a separate coding system. The purpose is to document pathological changes leading to partial or total condemnation of the carcass, and the findings are stored in the NFSA’s meat inspection case file system (MAKKS) from which we retrieved the “PM dataset”. If a carcass is redirected directly to the side rail without prior assessment of findings on the main slaughter line, EDR findings may be recorded at this stage.

As of 2025, the PM coding system for pigs included 49 different codes. Of these, 35 codes were presented in the PM dataset used in this study. According to NFSA instructions, partial condemnations are made only when the pathological finding affects more than 10% of the carcass weight [25]. The main finding is recorded as the reason behind the condemnation, but multiple additional findings can also be recorded. The meat inspection process is illustrated in Fig. 1.

### Data management

**Fig. 2:**
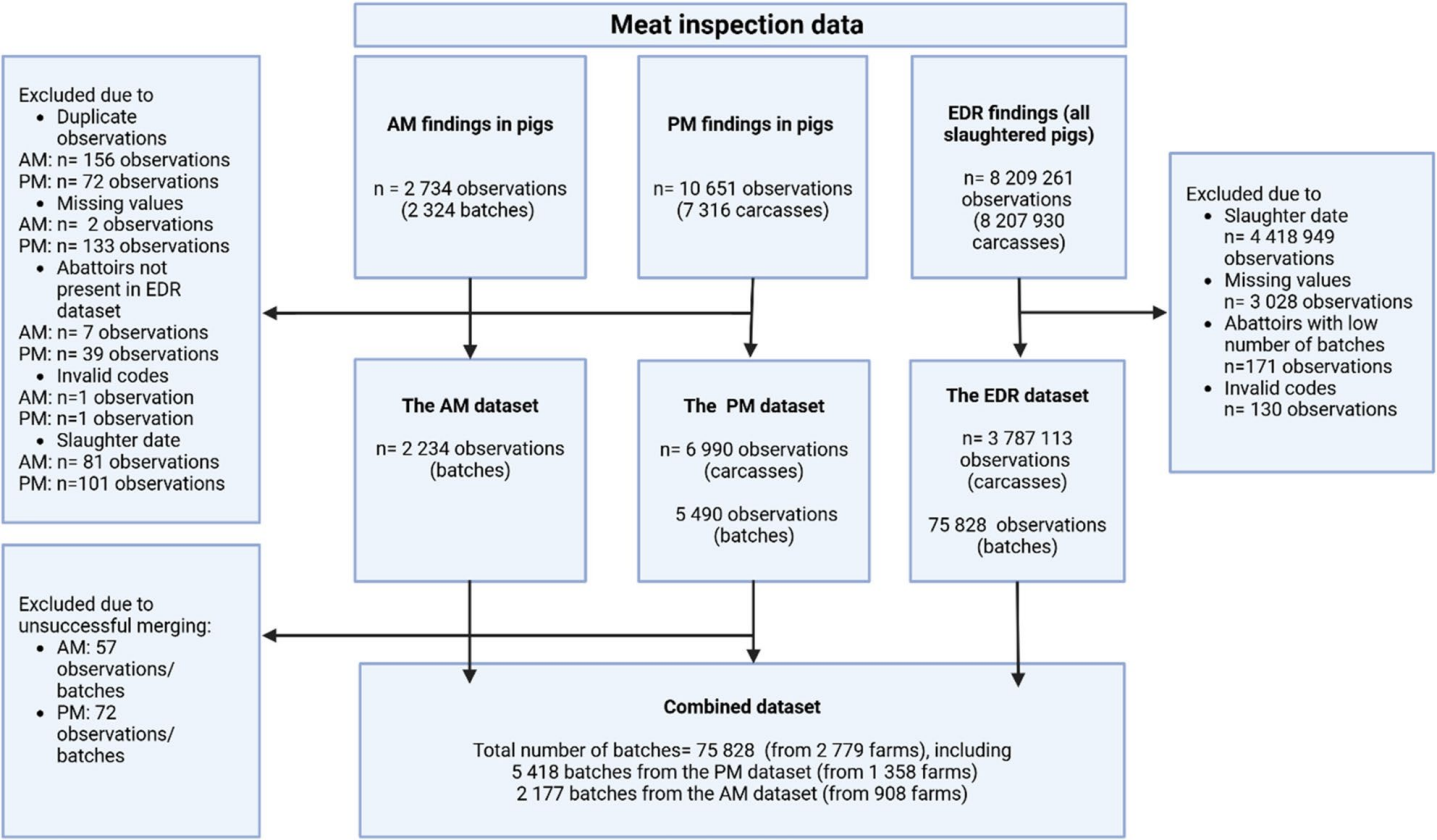
Flowchart of data cleaning for ante-mortem (AM), extended disease registration (EDR), and post-mortem (PM) datasets. Created in BioRender.com

During data cleaning, duplicate observations and records with missing values necessary for further analysis (e.g. missing farm ID) were removed from each dataset. Records with obvious errors (e.g. invalid codes for pigs) were disregarded. A detailed description of the data cleaning process is provided in Fig. 2. 

All three datasets shared the following common variables: a unique identification number (ID) for each abattoir and farm, date of slaughter, and animal category (fattening pig, sow and boar). All three datasets contained information about carcass condemnation, although the variable names and coding differed between the datasets. Additionally, each dataset included meat inspection findings specific to the inspection phase it represented. The EDR and PM datasets also contained carcass-level IDs. The batch ID was generated using the abattoir ID, farm ID and slaughter date.

In the AM dataset, we generated variables for the number of AM findings per batch and the number of pigs condemned per batch following AM inspection.

The EDR dataset was originally at the pig level, with one record per pig, regardless of whether a finding was detected. For the purpose of this study, data were aggregated to batch level. Some abattoirs (*n* = 4) outsource pig slaughtering to other facilities. For these cases, the abattoir ID was replaced with the abattoir ID of the facility where the slaughter took place. The following batch-level variables were created: number of pigs per batch; EDR findings per batch (count and proportion for each code); total EDR findings per batch (sum and proportion across all codes); slaughter year, slaughter month, slaughter week, day of week, and season (winter [Dec-Feb], spring [Mar-May], summer [Jun-Aug ] and autumn [Sep-Nov ]). A regional variable was also created, assigning all farms to one of five regions: Northern, Mid, Western, Southern, and Eastern Norway.

The PM dataset included individual pig carcass records. Cross-referencing carcass IDs with the EDR datasets identified eight discrepancies in slaughter dates, which were corrected to match the EDR records. After aggregating PM data to batch level, variables were created for the count of each main cause of condemnation, each PM diagnosis code, and condemned pigs per batch.

Data management and calculation of descriptive statistics were performed in Stata 18.0. after which the datasets were imported to R for further statistical analysis.

### Combining datasets

To maximize the usability of abattoir data, we aimed to link animals, batches and farms across the three datasets. Due to inconsistencies in carcass IDs, merging the PM and EDR dataset at the carcass level was not possible. Instead, we merged aggregated batch-level data using the generated batch ID. Non-matching batches were often due to initial discrepancies in abattoir IDs between dataset due to the ID not adequately reflecting the physical location of slaughter (see “Data management” section). Remaining unmatched batches were primarily due to discrepancies in slaughter dates. To address this, we allowed for a difference of -1 to + 7 days between recorded slaughter dates in the PM and the EDR datasets, using the EDR dataset as the reference.

Finally, the combined EDR and PM dataset were merged with the AM dataset, allowing for a one-day margin of discrepancy in slaughter data as AM inspections may occur the day before slaughter [3].

### Descriptive statistics

The frequency of each meat inspection finding was calculated as the number of affected carcasses or batches divided by the total examined. For AM findings, frequencies were calculated at the batch level; for EDR and PM findings, at the carcass level. For EDR findings we also calculated the 25th, 50th (median), 75th and 99th percentiles of the within-batch prevalence, defined as the proportion of positive pigs in each batch. The frequency of batches with and without EDR findings was also calculated for each animal category (finishing pigs, sows and boars). PM frequencies were expressed per 10 000 pigs, due to their low occurrence. Batch-level statistics were not calculated for the PM findings, as the PM dataset only included condemned carcasses. We also report the percentage of batches with at least one finding in the AM, EDR, or PM datasets. The proportion of batches with at least one finding (EDR and PM) was calculated per abattoir and visualised in bar charts. In addition, we calculated the number of farms and batches slaughtered per abattoir, and the number of abattoirs used by each farm.

### Variance partitioning analyses

To investigate the proportion of total variance in disease findings attributable to farms and abattoirs, we fitted binomial logistic mixed-effects models to the seven most common EDR and PM findings leading to carcass condemnation. The outcome variable was the frequency (count) of each disease finding per batch. For the EDR findings, we fitted models for the following seven outcomes: “abscesses and infected wounds”; “arthritis and joint lesions”; “pericarditis and/or pleuritis”; “pneumonia”; “ascariasis”; “healed tail lesions and short tails”; and “open tail wounds”. “Shoulder wounds” were excluded due to limited data. For the PM findings we fitted models for the following seven outcomes: “abscesses/phlegmons”; “arthritis”; “pneumonia”; “pleuritis”; “systemic disease”; “peritonitis”; and “gastrointestinal disease”. Because multiple disease findings could be recorded for a single pig, individual pigs may appear in the data for more than one of the modelled disease outcomes.

The statistical approach followed the methodology proposed for slaughterhouse surveillance data by Denwood et al. [28], which has been applied for abattoir data in both Denmark and Sweden [14, 15]. The response variable *Y*_*i*_ (i.e., the number of carcasses with a specific finding for batch *i*) was described using a binomial distribution:

$${Y}_{i} \sim Binomial\left({p}_{i},{N}_{i}\right)$$

where *p*_*i*_ is the probability of observing the outcome in batch *i*, and *N*_*i*_ is the total number of animals in that batch. The model was fitted under the assumption that the probability of recording a specific finding in a slaughter batch depends on both farm-specific characteristics and the abattoir where the meat inspection was conducted. Because farms may supply animals to multiple abattoirs and abattoirs receive animals from multiple farms, observations were cross-classified by farm and abattoir. To account for this, the model included a common intercept and cross-classified random effects for farm and abattoir. To account for overdispersion due to zero-inflated data, a random effect at batch-level was also included, as suggested by Browne et al. [29] and Harrison [30]. The general form of the model is shown in Eq. 1:

1 $$logit\left({p}_{i}\right)={\beta}_{0}+{A}_{m}+{F}_{n}+{B}_{i}$$

Where ${\beta}_{0}$ is the overall intercept, *A*_*m*_ is the random effect of abattoir *m*, *F*_*n*_ the random effect of farm *n* and *B*_*i*_ the random effect of batch *i*.

All models were fitted using the glmmTMB function of the “glmmTMB” package [31] in R version 4.4.3 [32].

The percentage of total variance attributable to abattoirs (VPC_*A*,_, Eq. 2) and farms (VPC_*F*_, Eq. 3) for each of the 14 models was calculated using variance partition coefficients (VPCs),

2 $${VPC}_{A}=\frac{{{\upsigma}}_{abattoir}^{2}}{{\sigma}_{abattoir}^{2}+{\sigma}_{farm}^{2}+{\sigma}_{batch}^{2}+{\sigma}_{\epsilon}^{2}}*100$$

3 $${VPC}_{F}=\frac{{{\upsigma}}_{farm}^{2}}{{\sigma}_{abattoir}^{2}+{\sigma}_{farm}^{2}+{\sigma}_{batch}^{2}+{\sigma}_{\epsilon}^{2}}*100$$

where ${{\upsigma}}_{abattoir}^{2}$ and ${{\upsigma}}_{farm}^{2}$ are the variance of random effects at abattoir and farm level, respectively, estimated from the model. ${\sigma}_{batch}^{2}$ is the overdispersion parameter (variation at batch-level) and ${\sigma}_{\epsilon}^{2}$ is the residual variance. The residual variance was estimated using the latent variable method [33], based on the assumption that each carcass has a specific likelihood of exhibiting a particular disease finding. Only carcasses whose likelihood surpasses a certain threshold are recorded with the finding. This approach assumes that the unobserved individual variable follows a logistic distribution with individual level variance equal to π^2^/3.

VPCs quantify the proportion of variation attributable to each level. A large VPC_*A*_ indicates substantial variation between abattoirs in the probability of recording a given finding, potentially affecting reliability. A VPC_*A*_ value close to zero indicates minimal variation between abattoirs. The VPC_A: F_ ratio can be used to assess whether variability at the abattoir level is similar to, or exceeds, that at the farm level.

To assess if variance components at the abattoir-level and farm-level change when additional contextual factors are considered, models were also run with season as fixed effect. Incorporating fixed independent variables reduces the risk of overestimating the variance components and the residual variance [34]. Seasonal variation in true prevalence is plausible for some diseases and if the temporal distribution of slaughtering also varies between abattoirs this may influence abattoir-level variation. If seasonal effects influence the variance components, attributing this variation solely to lack of agreement between abattoirs would be misleading. Region was also considered for inclusion given that spatial variation in true prevalence may influence variance components. However, due to multicollinearity with abattoir, region was omitted from the analysis.

To assess variation in disease detection across slaughter days, we introduced day of week as a fixed effect to the models. True disease prevalence is not expected to vary by day of week; therefore, an observed effect may indicate recording inconsistencies and poor data quality. Due to fewer batches slaughtered on weekends, Saturdays and Sundays were excluded from this analysis.

As the inclusion of fixed effects alter both the random effect variances (σ) and the total variance, the VPCs are not directly comparable between models. To evaluate the impact of including fixed effects variables, we report the VPC_A: F_ ratio and the direct difference in variance components (Δσ^2^) between models with and without fixed effects. This approach highlights changes in the random effects, independent of shifts in the total variance. Changes in variance at the abattoir (Eq. 4) and farm levels (Eq. 5) were calculated by subtracting the variance from models with fixed effects from the variance in the models with random effects only using the following equations:

4 $$\begin{aligned}\varDelta{\sigma}_{abattoir}^{2}&={\sigma}_{abattoir,\,random\,effects\,only}^{2}\\&-{\sigma}_{abattoir,\,models\,including\,fixed\,effects}^{2}\end{aligned}$$

5 $$\begin{aligned}\varDelta{\sigma}_{farm}^{2}&={\sigma}_{farm,\,random\,effects\,only}^{2}\\&-{\sigma}_{farm,\,models\,including\,fixed\,effects}^{2}\end{aligned}$$

$\varDelta{\sigma}_{abattoir}^{2}$ represents the change in abattoir-level variance, while $\varDelta{\sigma}_{farm}^{2}$ represents the change in farm-level variance attributable to the inclusion of fixed effects.

For meat inspection findings with a VPC_A: F_ ratio higher than one, we conducted sensitivity analyses to assess whether the observed variance components were driven by a few abattoirs with disproportionately high proportions of recorded findings. For each relevant finding, we identified abattoirs with the highest proportion of records and sequentially excluded them from the dataset. The models were then re-run to assess the changes in the variance components and the VPC_A: F_ ratios.

## Results

### Challenges in combining datasets

Individual pig-level merging was not feasible, primarily due to inconsistent carcass IDs between datasets, which resulted incomplete merging. Batch-level merging of the datasets was successfully achieved after extensive data wrangling. As all slaughtered pigs are recorded in the EDR dataset, all batches in the AM and PM datasets are expected to be present in the EDR dataset. We were able to merge 5418/5490 batches from the PM dataset and 2217/2234 batches from the AM dataset with the EDR dataset.

### Meat inspection findings for slaughtered pigs in Norway

During the 2.5-year study period, a total of 3 787 113 pigs from 75 828 batches and 2 779 farms were slaughtered at the 15 Norwegian abattoirs. Most farms (75.5%) sent their animals to a single abattoir, while 19.6% used two different abattoirs, and 4.9% sent animals to three to five abattoirs. The proportion of batches with at least one EDR or PM finding is visualised in Figure S1and S2, and the number of abattoirs used by each farm is shown in Table S1 (Supplementary material).

#### AM inspection

AM meat inspection resulted in at least one finding in 2.9% (2234/75 828) of all slaughtered batches. The median number of AM findings per batch was one, with a range of one to four. The most frequent AM findings were “clinical signs affecting animal welfare”, “severe tail wounds” and “dirty animals” (manure covering 20–50% of the body). In total, 0.007% pigs (254/3 787 367) were condemned prior to slaughter. The most common cause of condemnation pre-slaughter was “clinical signs of systemic disease”, accounting for 94.1% of the recorded causes at the individual pig level. Table 2 summarizes the frequency of AM findings recorded in more than 5% of the batches in the AM dataset. Complete frequency tables of all AM findings and explanations of all 17 AM codes can be found in Table S2 and S3 in the Supplementary material.

**Table 2:** Batch-level ante-mortem (AM) findings in 2 234 Norwegian pig batches

AM finding*	Number of batches with at least one finding	Overall frequency (%) of finding**
Clinical findings relevant to welfare	578	0.76
Severe tail wounds on multiple pigs	569	0.75
Other welfare-related conditions***	216	0.28
Dirty animals, 20–50% of body surface	205	0.27
Clinical signs of systemic disease	175	0.23
Housing-related injury sustained on farm	167	0.22
Missing identification	133	0.18
Total batches with ≥ 1 finding (any cause)	2234	2.95

*Only ante-mortem (AM) findings recorded in more than 5% of the batches are included

**Overall frequency is calculated by dividing the number of batches with at least one finding by the total number of batches slaughtered (*n* = 75 828)

***Conditions that may affect animal health and/or welfare, such as markedly agitated or stressed animals, excessive slap marks, or batches with uniformly short tails

#### PM inspection

PM meat inspection resulted in at least one recorded EDR finding in 15.6% (590 256/3 787 113) of all slaughtered carcasses across the study period. The median number of EDR findings per pig was zero, with range zero to six. “Ascariasis” was the most frequent finding, followed by “pericarditis and or/pleuritis”, and “abscesses and infected wounds” (Table 3).

**Table 3:** Carcass-level frequencies and within-batch percentiles for extended disease registration (EDR) findings in Norwegian pigs

EDR finding	Carcass level		Within-batch prevalence			
	Number	Percentage*	Percentiles (%)**			
			25th	50th	75th	99th
Ascariasis	265 813	7.02	0	0	6.67	73.33
Pericarditis and/or pleuritis	195 800	5.17	0	0	6.45	50.00
Abscesses and infected wounds	60 451	1.60	0	0	1.98	33.33
Short tails/ healed tail wound	53 301	1.41	0	0	1.11	25.00
Pneumonia	47 316	1.25	0	0	0	20.00
Open tail wound	37 288	0.98	0	0	0	16.67
Arthritis	20 622	0.54	0	0	0	13.0
Shoulder ulcers	942	0.02	0	0	0	0.71

* Overall frequency was calculated as the number of carcasses with the finding divided by the total number of slaughtered pigs across 15 abattoirs (*n* = 3 787 113)

** Percentiles of within-batch prevalence (percentage of carcasses with the finding per batch)

The median batch size was 32 pigs (interquartile range (IQR): 1-413; range 1-713). At batch level, 77.6% (58 835/75 282) of batches had at least one carcass with one or more EDR findings. There was considerable variability in the within-batch prevalence of different findings as shown by the percentiles in Table 3. For all findings, the median apparent prevalence was 0%, and the distributions were right-skewed, with some batches exhibiting notably higher proportions of positive findings. This was most pronounced for “ascariasis”, where the 99th percentile was 73% positive pigs.

Finishing pigs were the predominant animal category, comprising over 96% of slaughtered pigs. The proportion of carcasses with at least one recorded EDR finding among finishers was similar to boars and higher than sows (Table 4). Table 4 presents the frequency of carcasses with at least one recorded EDR finding by animal category.

**Table 4:** Frequency of carcasses with and without extended disease registration (EDR) findings by animal category

Animal category	EDR-		EDR+		Total
	Frequency	Percentage	Frequency	Percentage	*n*
Sow	107 711	88.2%	14 368	11.8%	122 079
Boar	11 803	85.0%	2 082	15.0%	13 885
Finishing pig	3 077 073	84.3%	574 076	15.7%	3 651 149
**Total**	3 196 587	84.4%	590 526	15.6%	3 787 113

EDR + = at least one EDR finding, EDR- = no EDR finding

Further post-mortem examination of carcasses placed “under arrest” led to partial or total condemnation in 0.18% (6990/3 787 113) of slaughtered carcasses. Among condemned carcasses the median number of PM findings per pig were one, with range one to seven. Of the 35 codes present in the dataset, 31 were assigned as the main cause of condemnation. The most frequent causes of condemnation were “systemic disease”, followed by “abscesses/phlegmons” and “gastrointestinal disease”. Table 5 shows the main causes of condemnation, including the frequencies of different findings for both total and partial condemnation.

**Table 5:** Main causes of post-mortem (PM) carcass condemnations in Norwegian pigs

PM finding*	All condemned**		Total condemnation***		Partial condemnation***	
	*n*	Per 10 000 pigs	*n*	%	*n*	%
Systemic disease	1 984	5.24	1984	30.22	0	0.00
Abscesses/phlegmons	803	2.12	589	8.97	214	50.47
Gastrointestinal disease	667	1.76	666	10.14	1	0.24
Arthritis	639	1.69	512	7.80	127	29.95
Peritonitis	559	1.48	556	8.47	3	0.71
Pneumonia	382	1.01	379	5.77	3	0.71
Pleuritis	359	0.95	338	5.15	21	4.95
Other codes	1 597	4.22	1 542	23.48	55	12.97
**Total in dataset**	**6 990**	**18.46**	**6 566**	**100**	**424**	**100**

* Only findings with a frequency > 5% in the PM dataset are included

**Overall frequencies were calculated as the number of carcasses with each finding divided by the total number of slaughtered pigs (*n* = 3 787 113) and expressed per 10 000 pigs

***Columns for total and partial condemnations show the number and percentage of carcasses totally or partially condemned for each finding

Among condemned carcasses, total condemnation was far more common (94%) compared to partial condemnation (6%). The reasons for condemnation differed markedly between total and partial cases: total condemnation was most often due to “systemic disease”, while “abscesses/phlegmons” and “arthritis” were the most common for causes of partial condemnation. A complete list of all main condemnation causes and explanations of the 35 PM codes used can be found in Table S4 and S5 (Supplementary material).

### Variance partitioning of EDR and PM findings

The variation between farms was higher than the variation between abattoirs for all EDR findings (VPC_A: F_ ratio < 1). “Ascariasis”, “open tail wounds” and “pericarditis and/or pleuritis” had the lowest VPC_A: F_ ratios, each below 0.1. The lowest between-abattoir variation was observed for pericarditis and/or pleuritis and open tail wounds (VPC_A_<1%). The variation for the remaining five other findings was also considered relatively low (VPC_A_<5%). The variance partitioning results for EDR findings from models with only random effects are presented in Table 6.

**Table 6:** Variance partitioning of seven extended disease registration (EDR) findings in Norwegian pigs

EDR finding	VPC_A_ (%)	VPC_F_ (%)	VPC_A: F_ (ratio)
Ascariasis	2.23	39.75	0.06
Pericarditis and/or pleuritis	0.98	22.84	0.04
Abscesses and infected wounds	2.56	4.44	0.58
Short tails/healed tail wounds	3.58	10.57	0.34
Pneumonia	2.52	11.90	0.21
Open tail wounds	0.50	11.73	0.04
Arthritis	4.41	6.75	0.65

Variance partition coefficients (VPC) were estimated using binomial logistic mixed models with random effects for abattoir and farm. VPC_A_ (%) shows the proportion of total variance attributable to abattoir, VPC_F_ (%) to farm, and the VPC_A: F_ ratio is the ratio between the two proportions

Among PM findings, there were notable differences in the distribution of variation. “Abscesses/phlegmons”, “systemic disease”, and “gastrointestinal disease” showed the highest variation between abattoirs, with VPC_A_ values exceeding 20%. For these findings, the variation between abattoirs was greater than the variation between farms (VPC_A: F_ ratio > 1). Conversely, the remaining PM findings showed negligible variation between abattoirs, with VPC_A_ values near 0%. “Peritonitis” showed negligible variation at both abattoir and farm levels. “Arthritis”, “pneumonia”, and “pleuritis” showed low variation overall, with slightly greater variation between farms than between abattoirs. The variance partitioning results for recordings of PM findings from models with random effects are presented in Table 7.

**Table 7:** Variance partitioning of the seven most frequent recorded post-mortem (PM) findings in Norwegian pigs

PM finding	VPC_A_ (%)	VPC_F_ (%)	VPC_A: F_ (ratio)
Systemic disease	21.84	5.93	3.68
Abscesses/phlegmons	24.93	4.15	6.01
Gastrointestinal disease	20.14	4.01	5.02
Arthritis	< 0.01	1.88	< 0.01
Peritonitis	< 0.01	< 0.01	< 0.01
Pneumonia	< 0.01	0.81	< 0.01
Pleuritis	< 0.01	1.30	< 0.01

Post-mortem (PM) findings from 15 Norwegian abattoirs, sorted by the frequency of each finding. Variance partition coefficients (VPC) were estimated using binomial logistic mixed models with random effects for abattoir and farm. VPC_A_ (%) shows the proportion of total variance attributable to abattoir, VPC_F_ (%) to farm, and the VPC_A: F_ ratio is the ratio between the two proportions

Excluding the most influential abattoirs for findings with a VPC_A: F_ ratio greater than one had variable influence on the variance components ($\varDelta{\sigma}_{abattoir}^{2}\mathrm{a}\mathrm{n}\mathrm{d}\varDelta{\sigma}_{farm}^{2})$, as well as on the VPC_A: F_ ratios. For systemic disease, one abattoir accounted for a large proportion of findings. Excluding this abattoir reduced the abattoir-level variance ($\varDelta{\sigma}_{abattoir}^{2}=0.49$), whereas the effect on farm level variance was negligible ($\varDelta{{\upsigma}}_{\mathrm{f}\mathrm{a}\mathrm{r}\mathrm{m}}^{2}=-0.01)$, and the VPC_A: F_ declined from 3.68 to 2.42. For “abscesses/phlegmons” two abattoirs accounted for the majority of findings. Sequential exclusion showed that removing the most influential abattoir reduced the abattoir-level variance ($\varDelta{\sigma}_{abattoir}^{2}=0.58)$, with minimal change of farm level variance ($\varDelta{{\upsigma}}_{\mathrm{f}\mathrm{a}\mathrm{r}\mathrm{m}}^{2}=0.01$), and the VPC_A: F_ declined from 6.01 to 3.84. Exclusion of both abattoirs simultaneously was not performed, as this would have left very few cases in the dataset. For gastrointestinal disease, exclusion of the most influential abattoir led to a marked reduction in abattoir-level variance ($\varDelta{\sigma}_{abattoir}^{2}=1.11)$, an increase in farm-level variance ($\varDelta{{\upsigma}}_{\mathrm{f}\mathrm{a}\mathrm{r}\mathrm{m}}^{2}=-1.25)$, and the VPC_A: F_ ratio dropped to near zero. The results for different subsets are presented in more detail in Table S6 (Supplementary material).

Including season as fixed effect had negligible influence on the variance components at both abattoir and farm level ($\varDelta{\sigma}_{abattoir}^{2},\varDelta{\sigma}_{farm}^{2})$ for all findings accounting for less than 0.01 of the total variances. Consequently, changes VPC ratios (VPC_A: F_) were also minimal. The variance components, VPC_A: F_ ratios, and changes when including season as fixed effect, can be found in Table S7 in the Supplementary material.

Regarding differences in detection of EDR findings between days, we found that day of week had a negligible effect on the apparent disease frequency across different findings (maximum OR: 1.12, with 95% CI: 1.05–1.19), indicating that meat inspection procedures did not vary substantially depending on the day of week. For PM findings, day of week also had minimal impact, with a statistically significant effect observed only for the PM finding gastrointestinal disease (OR: 1.3, 95% CI: 1.01–1.67).

## Discussion

In this study, we found that EDR findings were consistently detected across abattoirs, with greater variation at the farm level than at the abattoir level. This suggests that farm-related factors, such as herd health, management practices, and environmental conditions, are more important than abattoir differences in explaining variation. In contrast, certain PM findings - notably “systemic disease”, “abscesses/phlegmons” and “gastrointestinal disease” – showed considerable abattoir-level variation, likely reflecting differences in recording practices rather than in the true prevalence. The use of precise, standardized EDR definitions likely limits subjective interpretation. Conversely, the broader and less specific PM codes permit greater subjectivity, leading to increased variability in how findings are recorded.

Combining AM, PM and EDR datasets was challenging, due to inconsistencies in carcass IDs, recorded slaughter dates and abattoir IDs, mainly resulting from manual data entry of AM and PM findings and limitations in the NFSA’s case management system (personal communication with the NFSA, December 2024). These issues hamper traceability and highlight a need for harmonized, consistent documentation.

International comparison of meat inspection findings is challenging due to differences in coding systems and recording practices [13], and in study designs and study populations. For example, some studies include only certain categories of pigs, such as finishing pigs, sows, or in Italy, heavy pigs slaughtered at over 160 kg [5, 35]. The Norwegian dual coding system (EDR and PM) also complicates direct comparison. In this study, “ascariasis”, “pericarditis and/or pleuritis”, and “abscesses and infected wounds” were the most common EDR findings, consistent with reports from other European studies, though the rank and apparent prevalences vary [5, 11, 14, 16, 36–38]. While pulmonary disease is one of the most common findings in several European studies [11, 38, 39] this was not observed in the present study. This might reflect improved respiratory health in Norwegian pigs, with documented freedom from several important respiratory pathogens, such as Porcine respiratory and reproductive syndrome virus (PRSSV) [40] and *Mycoplasma Hyopneumoniae* [41]. Additionally, different case definitions (for EDR: lung lesions > 2.5 cm equals positive finding, see Table 1) across different countries may also contribute to differences in reported prevalence.

PM condemnations were recorded for 0.18% of slaughtered pigs, with “systemic disease” and “abscesses/phlegmons” most frequent. These are apparent frequencies, influenced by the sensitivity and specificity of the inspection procedure, and missing data (e.g., not all animals sent for slaughter). However, estimating true prevalence was not the objective of this study. European studies report a wide range of condemnation frequencies and causes [5, 11, 13]. These variations are likely affected by differences in registration practices and/or true differences in prevalence. In Norway, a higher threshold for mandatory recording of partial condemnations likely contributes to the lower reported frequency.

Variance partitioning analysis showed consistently low abattoir-level variation for all EDR findings, especially for “pericarditis and/or pleuritis” and “open tail wounds”, suggesting uniform detection practices between abattoirs. In contrast, the three most common PM findings showed substantial abattoir-level variation, indicating they may be prone to inconsistencies in detection and classification. For the PM finding “gastrointestinal disease”, nearly all abattoir-level variation was attributed to a single abattoir. For “abscesses/phlegmons” and “systemic disease” abattoir-level variance persisted even after removing the most influential abattoirs (VPC_A: F_ ratio 3.8 and 2.4, respectively). However, some of the observed variation may also reflect spatial differences in true prevalence which could not be accounted for in our analyses.

The observed differences in abattoir-level variation between the EDR and certain PM findings likely reflect both differences in diagnostic criteria and the intended purpose of these systems. The EDR system captures a wide range of clinical findings using precise and standardized definitions and generally lower thresholds for recording, likely reducing subjectivity and variation between abattoirs. In contrast, PM findings are recorded only when condemnation is required, making them more dependent on individual judgement – especially where code definitions or diagnostic guidelines are unclear. This increases the risk of inconsistencies in detection and classification. Diagnosing certain findings, such as “gastrointestinal disease”, can be challenging, as meat inspection relies mainly on visual inspection, increasing the risk of misclassification. Additionally, limited exposure and training in assessing severe cases, as well as the economic consequences of condemnations, may influence inspectors’ threshold for condemnation.

Our results partly align with previous European studies employing variance partitioning, which found that the variation in PM findings can range from low to high, both between abattoirs [14, 15] and between inspectors within the same abattoirs [16]. The specific findings with the lowest variation, however, differ between studies [14–16]. For instance, Comin et al. [14] reported low between-abattoir variation for the presence of liver parasites and abscesses, but high for injuries and unspecific findings, suggesting that some findings may be more prone to subjective interpretation. In contrast, a Danish study reported substantial abattoir-level variation for most meat inspection findings, with only a few codes, such as abscesses and chronic arthritis, showing low variation [15]. The differences between studies, including the present, likely reflect differences in national coding systems and inspection practices (as discussed above), as well as differences in data structure. For example, in the Danish study by Nielsen et al. [15], a batch was defined as all pigs slaughtered at a specific abattoir from a specific farm during a given year, whereas in our study, a batch refers to pigs from the same farm, slaughtered at the same abattoir at the same date. Furthermore, the systematic recording and precise lesion definitions in the Norwegian EDR system may contribute to the lower variation observed for these findings compared to systems in other countries.

Season had minimal impact on the VPC_A: F_ ratio and variance components (Δσ^2^) at both abattoir and farm level for all findings, indicating that seasonal differences in slaughter frequencies did not affect the results. However, year was not included in the models, and each season was observed only a few times over the 2.5-year period, limiting our ability to distinguish year-to-year variation from seasonal effects.

In a previous study, Schleicher et al. [16] included inspectors as a random effect to estimate how much variation in meat inspection findings could be attributed to individual inspectors. The adding of quarter (three-month periods) and other fixed effects had little impact on inspector-level variance, but did explain some of the variance at the farm-level, thereby increasing the relative contribution of inspector-level variance [16]. In our study, inspector IDs were unavailable, and the variation attributable to inspector could therefore not be assessed.

Day of week had minimal to no effect for most findings, except for the PM finding “gastrointestinal disease” which showed a significant slaughter-day effect (OR: 1.3, 95% CI: 1.01–1.67). This suggests weekly variation, possibly due to operational differences (e.g., slaughter volume, batch composition and origin, inspection routines). As previously mentioned, there are inherent challenges in relying on visual assessment for this code. Our results pose concerns about the reliability of this PM finding and highlight the need for improved consistency before it can be used reliably for surveillance purposes.

While we have evaluated certain aspects of data quality (e.g., consistency in recording practices and dataset integration), this study did not comprehensively assess data accuracy or completeness. As highlighted by Birkegaard et al. [12], systematic evaluation of registry data should address validity, completeness, timeliness, simplicity, flexibility, and usefulness. A limitation to our analyses is that regional effects (e.g., climate, management, biosecurity) could not be accounted for, potentially leading to overestimation of abattoir-level variance. The lack of observer ID prevented direct assessment of inter-observer variance; thus, the abattoir random effect likely captures both between-inspector differences and other intra-abattoir factors (e.g., technical equipment, working conditions, and routines). Including observer ID in future recording systems would enable a more precise evaluation of both inter-observer variation and diagnostic accuracy.

If farmers selectively choose which abattoirs to send their animals to in different situations, such as sending compromised animals to specific abattoirs, this may introduce bias to the variance partitioning estimates. Furthermore, geographical differences in disease occurrence are plausible for some diseases and may contribute to the abattoir level variation in our study. Thus, some abattoir-level variance may reflect true differences in disease occurrence.

Although these limitations must be acknowledged, they are unlikely to undermine the overall validity of the study, which included all major Norwegian pig abattoirs. Our findings provide a robust overview of meat inspection practices across the Norwegian pig slaughter industry. Reducing abattoir-related variation is essential to ensure meat inspection data can support targeted efforts to improve animal health and welfare throughout the production chain. When such data reliably reflect farm-level conditions, producers, advisors, and abattoirs are better equipped to implement effective interventions and monitor progress over time. Advances in digitalization, such as computer vision systems for PM inspections, may further enhance standardization and objectivity [42, 43]. However, to realize the full potential of these systems, harmonized coding systems with clear case definition criteria are essential.

## Conclusion

This study demonstrated that EDR findings are consistently detected across abattoirs, supporting the potential of EDR data as a reliable source for animal welfare surveillance. However, considerable variation in certain PM findings suggests inconsistencies in recording practices between abattoirs. Season had minimal influence on the variance components, while day of week only affected variance for the PM finding gastrointestinal disease. The process of linking data throughout the slaughter chain revealed notable challenges, highlighting the need for harmonization to improve traceability. Future efforts should focus on standardizing recording procedures and improving data quality to fully realize the potential of meat inspection data for animal health and welfare surveillance in Norway.

## Supplementary Information

Supplementary Material 1. Supplementary material. Meat inspection of pigs slaughtered in Norwegian abattoirs: Insights from variance partitioning analysis. This file contains supplementary material for the manuscript, including two additional figures and seven tables referred to in the manuscript. These materials provide further details and results that support the findings presented in the study.

## Data Availability

The data used in this study were obtained from the Norwegian Food Safety Authority and are subject to restrictions. These data were used under license and are not publicly available. However, the data may be made available by the authors upon reasonable request and with permission of the Norwegian Food Safety Authority.
