# Supplementary material for: Meat inspection of pigs slaughtered in Norwegian abattoirs: insights from variance partitioning analysis
Source: BMC Vet Res. 2026 Mar 11;22:240. doi: 10.1186/s12917-026-05401-2 (PMC13088660; doi:10.1186/s12917-026-05401-2)
Supplement: Supplementary file 1 — Supplementary Material 1. Supplementary material. Meat inspection of pigs slaughtered in Norwegian abattoirs: Insights from variance partitioning analysis. This file contains supplementary material for the manuscript, including two additional figures and seven tables referred to in the manuscript. These materials provide further details and results that support the findings presented in the study. [file 12917_2026_5401_MOESM1_ESM.zip › Additional file 1.docx]

**Supplementary material**

**Meat inspection of pigs slaughtered in Norwegian abattoirs: Insights from variance partitioning analysis**

List of authors: Kristine Paulsen Eggen^1,2^, Hilde Vinje^3^, Camilla Kielland^1^, Marit Nesje^1^, Ingeborg Sveinsdottir^2,4^, Ingrid Toftaker^1^

^1^ Department of Production Animal Clinical Sciences, Faculty of Veterinary Medicine, Norwegian University of Life Sciences, Ås, Norway.

^2^ Division Land Animals and Abattoir, The Norwegian Food Safety Authority, Brumunddal, Norway

^3^ Faculty of Chemistry, Biotechnology and Food Science, Norwegian University of Life Sciences, Ås, Norway.

^4^ Section Terrestrial Animal Health and Welfare, The Norwegian Veterinary Institute, Ås, Norway

- *-*

**Supplementary figures and tables**

**Figure S1.** Proportion of batches with at least one post-mortem (PM) findings across 15 Norwegian abattoirs


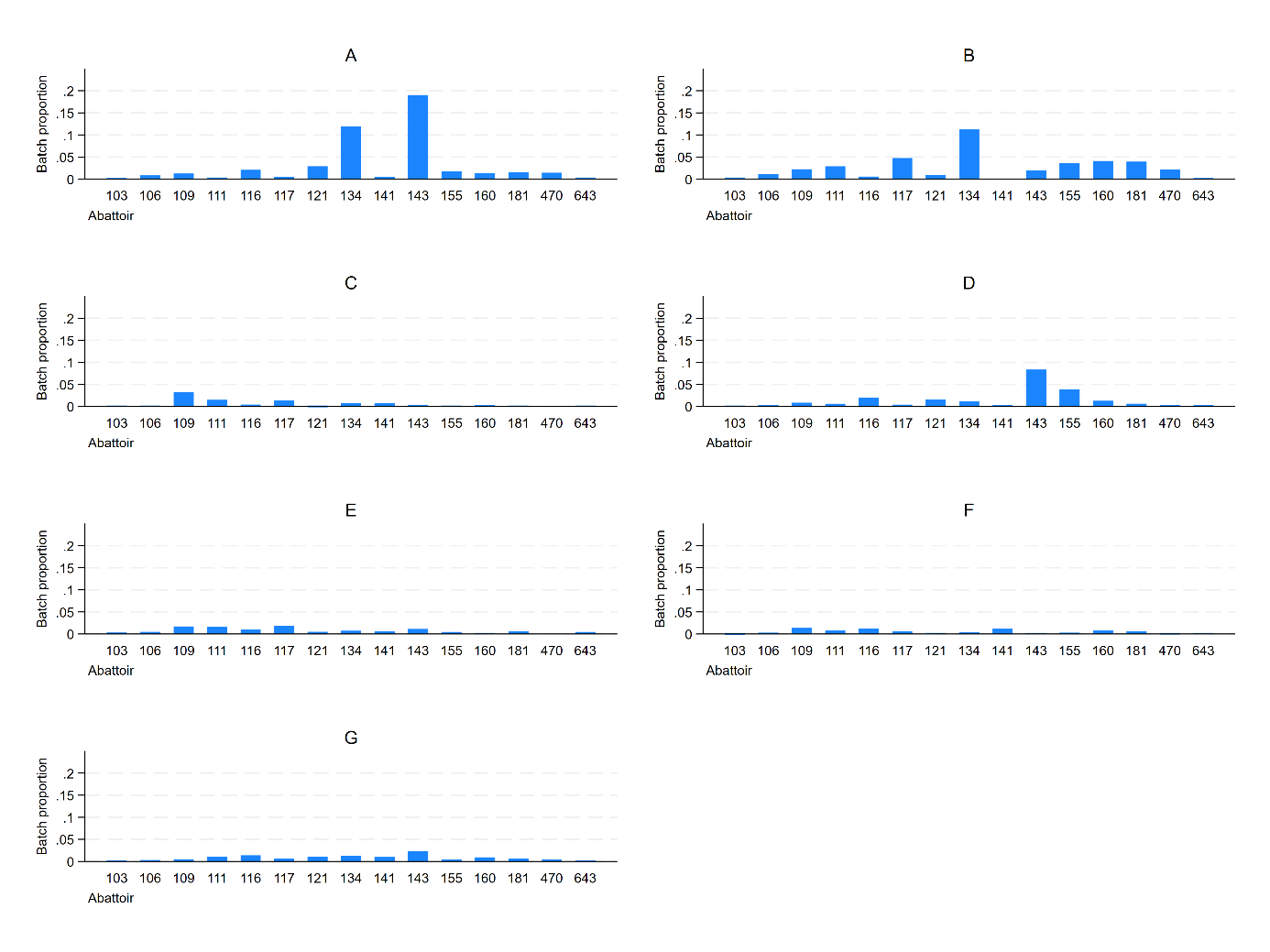
Bar charts showing the proportion of batches with at least one pig diagnosed with the seven most common post-mortem (PM) findings at 15 abattoirs (October 2021 to March 2024). Batch proportions are calculated as the number of batches with at least one case by the total number of batches slaughtered at each abattoir. Panels A-G: A: “abscesses/phlegmons”; B: “systemic disease”; C: “gastrointestinal disease”; D: “arthritis”; E: “peritonitis”; F: “pneumonia”; G: “pleuritis”

**Figure S2.** Proportion of batches with extended disease registration (EDR) findings across 15 Norwegian abattoirs, 2021-2024

**
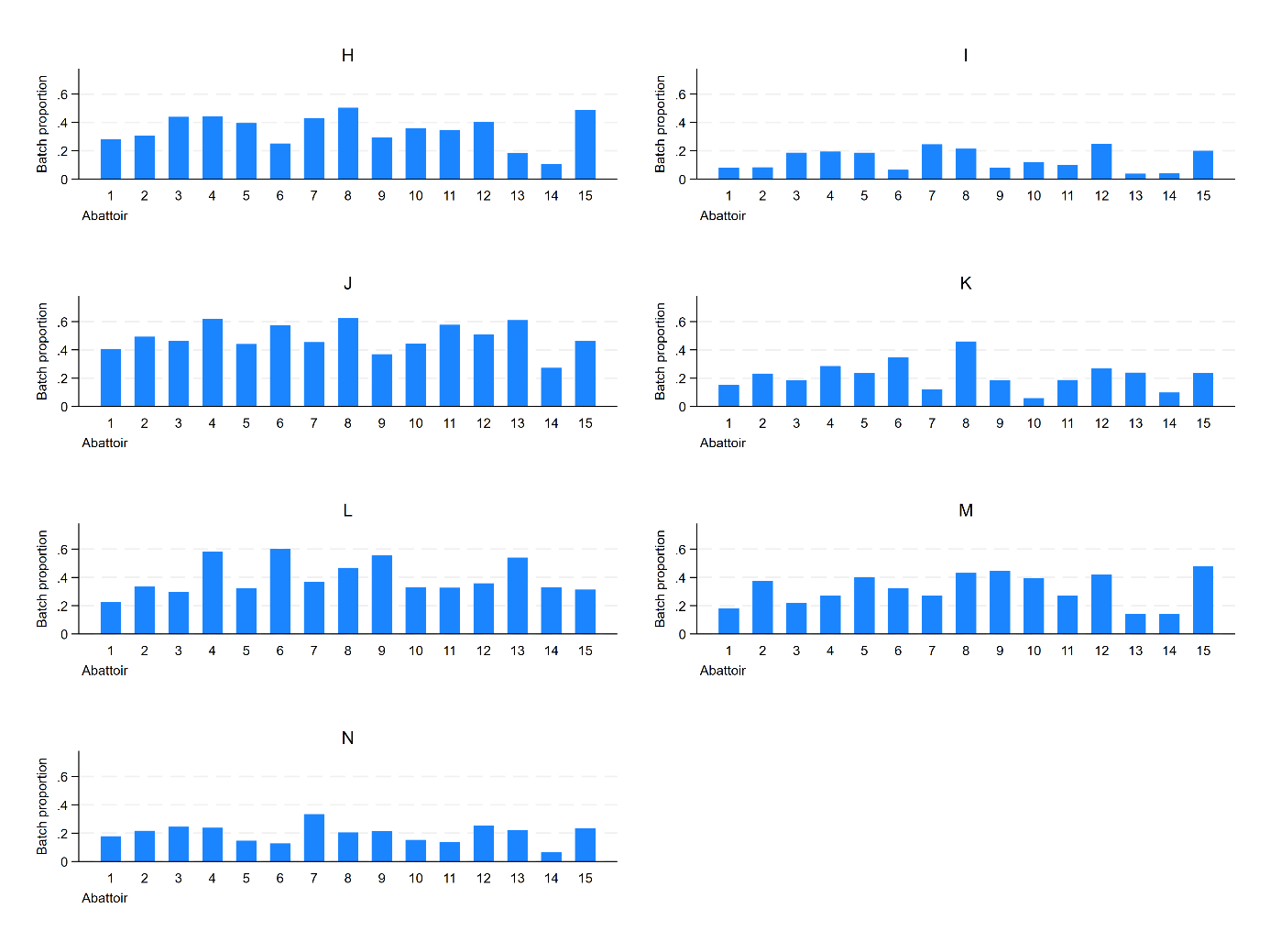
**Bar charts showing the proportion of batches with at least one pig diagnosed with the seven most common extended disease registration (EDR) findings at 15 abattoirs (October 2021 to March 2024). Batch proportions are calculated as the number of batches with at least one case by the total number of batches slaughtered at each abattoir. Panels H-N: H: “abscesses”; I: “arthritis”; J: “pericarditis and/or pleuritis”; K: “pneumonia”; L: “ascariasis”; M: “short/healed tails”; N: “open tail wounds”

**Table S1.** Frequency of abattoirs utilized by each farm

| **Number of different abattoirs utilized by one farm** | **Pig farms** | |
| --- | --- | --- |
|  | **Number** | **Percentage** |
| 1 | 2 099 | 75.53 |
| 2 | 545 | 19.61 |
| 3 | 113 | 4.07 |
| 4 | 21 | 0.76 |
| 5 | 1 | 0.04 |
| **Total** | **2 779** | **100.00** |

Data from 15 Norwegian Abattoirs, October 2021- March 2024

**Table S2.** Description of ante-mortem (AM) findings

| **AM Finding** | **Description** | **Decision*** |
| --- | --- | --- |
| Open tail wound | One or more pigs with open tail wounds likely originating on the farm. | 0 |
| Long claws | Abnormally long or deformed claws. | 0 |
| Poor body condition, category 1 | Hips and spine palpable with light pressure; spine barely visible from behind. Well-developed musculature. | 0/1 |
| Poor body condition, category 2 | Hips and spine visible; spine prominent from behind and sunken musculature. | 0/1 |
| Clinical sign of systemic disease | Clinical signs of ongoing infection (e.g., depression, fever). | 0/1 |
| Dirty animals, category 1 | Manure covering 20% -50 of the body, no or minor skin redness. | 0/1 |
| Dirty animals, category 2 | Manure covering > 50% of the body, or areas with a thick layer of manure; skin erosion of any size may be present. | 0/1 |
| Non-compliance with regulations for identification | Animals not identified in accordance with legal requirements. | 0/1 |
| Other housing injuries occurred on farm | Chronic conditions likely related to housing conditions, e.g., neck wounds, bursitis, shoulder wounds, or pressure sores. For shoulder wounds in sows, only grades 3 and 4 are recorded. | 0 |
| Unfit for transport | Phased out following updates to the coding framework. |  |
| Other welfare-related conditions | Other conditions potentially affecting animal health or welfare, e.g., markedly agitated or stressed animals, excessive slap marks or consignments with uniformly short tails (indicative of tail docking). | 0/1 |
| Clinical signs relevant to welfare | Findings significant to animal health, animal welfare, and fitness for food, e.g., lameness, eye disease, abscesses or swellings, wounds, or large hernia affecting transportability. | 0 |
| Ear notches | Multiple animals with similar ear notches in the outer ear. | 0 |
| Missing tails | Missing tails or only short stump | 0 |
| Growth disorders | Abnormal proportions for age/weight. | 0 |
| Residues / foreign substances – withdrawal period not expired | Veterinary drugs residues or foreign substances present; withdrawal period not expired. | 1 |

*Decision 0 = no consequences for food safety, Decision 1 = condemnation pre-slaughter

**Table S3.** Batch-level ante-mortem **(**AM) findings in pigs: 2 234 batches from 15 Norwegian abattoirs, October 2021-March 2024.

| **AM finding\|** | **Number of batches with at least one finding** | **Overall frequency (%) of finding*** |
| --- | --- | --- |
| Clinical signs relevant to welfare | 578 | 0.76 |
| Severe tail wounds | 569 | 0.75 |
| Other welfare-related conditions** | 216 | 0.28 |
| Dirty animals, category 1 | 212 | 0.27 |
| Clinical signs of systemic disease | 175 | 0.23 |
| Housing-related injury sustained on farm | 167 | 0.22 |
| Missing identification | 113 | 0.18 |
| Missing tails | 105 | 0.14 |
| Unfit for transport | 94 | 0.12 |
| Poor body condition, category 1 | 84 | 0.11 |
| Long claws | 56 | 0.07 |
| Eat notches | 36 | 0.05 |
| Poor body condition, category 2 | 28 | 0.04 |
| Dirty animals, category 2 | 22 | 0.03 |
| Acute injuries | 3 | <0.01 |
| Growth disorders | 2 | <0.01 |
| Residues/foreign substances | 2 | <0.01 |
| **Total number of findings** | **2 462***** |  |

* Overall frequency is calculated by dividing the number of batches with at least one finding by the total number of batches slaughtered (n=75 828).

** Conditions that may affect animal health and/or welfare, such as markedly agitated or stressed animals, excessive slap marks, or batches with uniformly short tails.

***The total number of findings exceeds the number of slaughtered batches, as multiple findings could be recorded for the same batch

**Table S4**. Post-mortem (PM) findings

| **PM finding** | **Decision*** |
| --- | --- |
| Abscesses/phlegmons | 1/2 |
| Arthritis | 1/2 |
| Pericarditis | 1/2 |
| Pneumonia | 1/2 |
| Pleuritis | 1/2 |
| Ante-mortem inspection not performed | 1 |
| Associated viscera not examined post-mortem | 1 |
| Dead before slaughtering, , stillborn, unborn, or slaughtered before 7 days of age. | 1 |
| Tuberculosis | 1/2 |
| Systemic disease (sepsis, pyaemia, toxaemia, or viraemia) | 1 |
| Traumatic soft tissue injuries | 1/2 |
| Bone fracture | 1/2 |
| Peritonitis | 1/2 |
| Marked deviation in odour or taste that persist after 24 hours (boar taint not included) | 1 |
| Marked deviation in colour (e.g., icterus, melanosis) | 1/2 |
| Insufficient bleeding | 1/2 |
| Emaciated animals | 1/0 |
| Parasitic disease | 1/2 |
| Residues of chemical substances or contaminants above permitted threshold | 1 |
| Foreign bodies | 1/2 |
| General circulatory disorder with ascites/oedema | 1 |
| Metabolic disease | 1/2 |
| Tumours | 1/2 |
| Skin disease (e.g., eczema and dermatitis, necrosis, burns, infected wounds, erysipelas, scabies, and exudative epidermitis in pigs) | 1/2 |
| Muscular disease | 1/2 |
| Central nervous system disease | 1 |
| Disease in heart, vessels, and pericardium (e.g., endocarditis) | 1/2 |
| Gastrointestinal disease (e.g., gastroenteritis, colic and ileus) | 1 |
| Urinary tract disease | 1/2 |
| Disease in the reproductive organs | 1/2 |
| Facal contamination | 1/2 |
| Meat containing blood posing health risks due to animal condition or slaughter contamination | 1/2 |
| Meat that may pose a health risk to humans or animals, or is deemed unsuitable for consumption | 1/2 |
| Not identified carcass | 1 |

*Decision 0 = no consequences for fitness for consumption/food safety, Decision 1 = total condemnation, Decision 2 = partly condemnation.

**Table S5.** The main causes of post-mortem (PM) condemnation in pigs in 15 Norwegian abattoirs, 2021-2024

| **PM finding** | **All condemned*** | |  | **Total condemnation**** | |  | **Partial condemnation**** | |
| --- | --- | --- | --- | --- | --- | --- | --- | --- |
|  | **n** | **Per 10 000 pigs** |  | **n** | **%** |  | **n** | **%** |
| Systemic disease | 1 984 | 5.24 |  | 1984 | 30.22 |  | 0 | 0.00 |
| Abscesses/phlegmons | 803 | 2.12 |  | 589 | 8.97 |  | 214 | 50.47 |
| Gastrointestinal disease | 667 | 1.76 |  | 666 | 10.14 |  | 1 | 0.24 |
| Arthritis | 639 | 1.69 |  | 512 | 7.80 |  | 127 | 29.95 |
| Peritonitis | 559 | 1.48 |  | 556 | 8.47 |  | 3 | 0.71 |
| Pneumonia | 382 | 1.01 |  | 379 | 5.77 |  | 3 | 0.71 |
| Pleuritis | 359 | 0.95 |  | 338 | 5.15 |  | 21 | 4.95 |
| Pericarditis | 284 | 0.75 |  | 284 | 4.33 |  | 0 | 0.00 |
| Emaciated animals | 253 | 0.67 |  | 253 | 3.85 |  | 0 | 0.00 |
| Abnormal colour | 229 | 0.60 |  | 229 | 3.49 |  | 0 | 0.00 |
| Disease in heart, vessels, and pericardium | 178 | 0.47 |  | 178 | 2.71 |  | 0 | 0.00 |
| Skin disease | 170 | 0.45 |  | 156 | 2.38 |  | 14.0 | 3.30 |
| Tumours | 143 | 0.38 |  | 143 | 2.18 |  | 0 | 0.00 |
| Tuberculosis | 100 | 0.26 |  | 100 | 1.52 |  | 0 | 0.00 |
| General circulatory disorder with ascites/edema | 63 | 0.17 |  | 63 | 0.96 |  | 0 | 0.00 |
| Urinary tract disease | 61 | 0.16 |  | 61 | 0.93 |  | 0 | 0.00 |
| Health risk | 45 | 0.12 |  | 45 | 0.69 |  | 0 | 0.00 |
| Faecal contamination | 17 | 0.04 |  | 3 | 0.05 |  | 14 | 3.30 |
| Traumatic soft tissue injuries | 12 | 0.03 |  | 0 | 0.00 |  | 12 | 2.83 |
| Bone Fracture | 8 | 0.02 |  | 0 | 0.00 |  | 8 | 1.89 |
| Dead before slaughter, stillborn, too young | 7 | 0.02 |  | 7 | 0.11 |  | 0 | 0.00 |
| Organs not examined PM | 6 | 0.02 |  | 6 | 0.09 |  | 0 | 0.00 |
| Abnormal odour/taste | 5 | 0.01 |  | 5 | 0.08 |  | 0 | 0.00 |
| Muscular disease | 4 | 0.01 |  | 1 | 0.02 |  | 3 | 0.71 |
| Foreign bodies | 3 | 0.01 |  | 0 | 0.00 |  | 3 | 0.71 |
| Inadequate bleeding | 2 | 0.01 |  | 2 | 0.03 |  | 0 | 0.00 |
| Genital disease | 2 | 0.01 |  | 1 | 0.02 |  | 1 | 0.24 |
| Contains blood that may pose a health risk | 2 | 0.01 |  | 2 | 0.03 |  | 0 | 0.00 |
| Metabolic disorders | 1 | <0.01 |  | 1 | 0.02 |  | 0 | 0.00 |
| Residues/contaminants | 1 | <0.01 |  | 1 | 0.02 |  | 0 | 0.00 |
| Not examined AM | 1 | <0.01 |  | 1 | 0.02 |  | 0 | 0.00 |
| **Total in dataset** | **6 990** | **18.46** |  | **6 566** | **100** |  | **424** | **100** |

*Overall frequencies were calculated as the number of carcasses with each finding divided by the total number of slaughtered pigs (n= 3 787 113) and expressed per 10 000 pigs. **Columns for total and partial condemnations show the number and percentage of carcasses totally or partially condemned for each finding.

**Table S6.**  Changes in variance components and VPC ratios across mixed models excluding the most influential abattoirs

| **PM finding,** | | **Random effects models** | | | **Exclusion of abattoir** | | |
| --- | --- | --- | --- | --- | --- | --- | --- |
|  |  | **Var_abattoir_** | **Var_farm_** | **VPC_A:F_** | $\boldsymbol{\Delta}\boldsymbol{\sigma}_{\boldsymbol{abattoir}}^{\boldsymbol{2}}$ | $\boldsymbol{\Delta}\boldsymbol{\sigma}_{\boldsymbol{farm}}^{\boldsymbol{2}}$ | **VPC_A:F_** |
| Systemic disease* | | 1.49 | 0.41 | 3.68 | 0.49 | -0.01 | 2.42 |
| Abscesses/  phlegmons |  | |  |  |  |  |  |
| Abattoir 10 | | 1.48 | 0.25 | 6.01 | 0.58 | 0.01 | 3.84 |
| Abattoir 8 | | 1.48 | 0.25 | 6.01 | 0.29 | -0.01 | 4.62 |
| Gastro-intestinal disease ** | | 1.11 | 0.22 | 5.02 | 1.11 | -1.25 | <0.01 |

The table presents the changes in residual variance (Δσ²) at the farm and abattoir level ($\Delta\sigma_{\mathrm{abattoirs}}^{2}, \Delta\sigma_{\mathrm{farm}}^{2}$), and the corresponding VPC ratios for the mixed models excluding the most influential abattoirs. Δσ² are the changes in variance components compared to the models with only random effects. Abattoir number reflects the numbering used in Figure 1. *Excluding abattoir 8, **excluding abattoir 3

**Table S7*.***  Changes in variance components and VPC ratios across mixed models with season as fixed effect.

| **EDR finding** | **Random effects models** | | | **Season as fixed effect** | | |
| --- | --- | --- | --- | --- | --- | --- |
|  | **Var_abbatoir_** | **Var_farm_** | **VPC_A:F_** | $\boldsymbol{\Delta}\boldsymbol{\sigma}_{\boldsymbol{abattoir}}^{\boldsymbol{2}}$ | $\boldsymbol{\Delta}\boldsymbol{\sigma}_{\boldsymbol{farm}}^{\boldsymbol{2}}$ | **VPC_A:F_** |
| Ascariasis | 0.20 | 3.60 | 0.06 | -0.02 | 0.06 | 0.06 |
| Pericarditis and/or pleuritis | 0.05 | 1.24 | 0.04 | <-0.01 | <-0.01 | 0.04 |
| Abscesses and infected wounds | 0.10 | 0.18 | 0.58 | <0.01 | <-0.01 | 0.57 |
| Short tails/healed tail wounds | 0.18 | 0.52 | 0.34 | <-0.01 | <-0.01 | 0.34 |
| Pneumonia | 0.14 | 0.68 | 0.21 | <-0.01 | <0.01 | 0.21 |
| Open tail wounds | 0.02 | 0.56 | 0.04 | <-0.01 | <0.01 | 0.05 |
| Arthritis | 0.21 | 0.32 | 0.65 | <0.01 | <0.01 | 0.65 |
| **PM finding** |  |  |  |  |  |  |
| Systemic disease | 1.49 | 0.41 | 3.68 | -0.02 | <0.01 | 3.72 |
| Abscesses/  phlegmons | 1.48 | 0.25 | 6.01 | <-0.01 | <-0.01 | 5.99 |
| Gastro-intestinal disease | 1.11 | 0.22 | 5.02 | <0.01 | <0.01 | 5.02 |
| Arthritis | <0.01 | 1.52 | <0.01 | <-0.01 | <-0.01 | <0.01 |
| Peritonitis | <0.01 | <0.01 | <0.01 | <-0.01 | <-0.01 | <0.01 |
| Pneumonia | <0.01 | 0.74 | <0.01 | <0.01 | <-0.01 | <0.01 |
| Pleuritis | <0.01 | 1.16 | <0.01 | <-0.01 | <-0.01 | <0.01 |

The table presents the changes in residual variance (Δσ²) at the farm and abattoir level ($\Delta\sigma_{\mathrm{abattoirs}}^{2}, \Delta\sigma_{\mathrm{farm}}^{2}$), and the corresponding VPC ratios for the mixed models including season as fixed effect for each EDR and PM finding. Δσ² represents the change in variance compared to the models with only random effects.
