# Supplementary material for: Meat inspection of pigs slaughtered in Norwegian abattoirs: insights from variance partitioning analysis
Source: BMC Vet Res. 2026 Mar 11;22:240. doi: 10.1186/s12917-026-05401-2 (PMC13088660; doi:10.1186/s12917-026-05401-2)
Supplement: Supplementary file 1 — Supplementary Material 1. Supplementary material. Meat inspection of pigs slaughtered in Norwegian abattoirs: Insights from variance partitioning analysis. This file contains supplementary material for the manuscript, including two additional figures and seven tables referred to in the manuscript. These materials provide further details and results that support the findings presented in the study. [file 12917_2026_5401_MOESM1_ESM.zip › Additional file 2, supplementary table and figure captures.docx]

**Supplementary Figure and Table Captures**

**Figure S1.** Proportion of batches with at least one post-mortem (PM) findings across 15 Norwegian abattoirs. Bar charts showing the proportion of batches with at least one pig diagnosed with the seven most common post-mortem (PM) findings at 15 abattoirs (October 2021 to March 2024). Batch proportions are calculated as the number of batches with at least one case by the total number of batches slaughtered at each abattoir. Panels A-G: A: “abscesses/phlegmons”; B: “systemic disease”; C: “gastrointestinal disease”; D: “arthritis”; E: “peritonitis”; F: “pneumonia”; G: “pleuritis”

**Figure S2.** Proportion of batches with extended disease registration (EDR) findings across 15 Norwegian abattoirs, 2021-2024. Bar charts showing the proportion of batches with at least one pig diagnosed with the seven most common extended disease registration (EDR) findings at 15 abattoirs (October 2021 to March 2024). Batch proportions are calculated as the number of batches with at least one case by the total number of batches slaughtered at each abattoir. Panels H-N: H: “abscesses”; I: “arthritis”; J: “pericarditis and/or pleuritis”; K: “pneumonia”; L: “ascariasis”; M: “short/healed tails”; N: “open tail wounds”

**Table S1.** Frequency of abattoirs utilized by each farm. Data from 15 Norwegian Abattoirs, October 2021- March 2024.

**Table S2.** Description of ante-mortem (AM) findings.

**Table S3.** Batch-level ante-mortem **(**AM) findings in pigs: 2 234 batches from 15 Norwegian abattoirs, October 2021-March 2024. * Overall frequency is calculated by dividing the number of batches with at least one finding by the total number of batches slaughtered (n=75 828).

** Conditions that may affect animal health and/or welfare, such as markedly agitated or stressed animals, excessive slap marks, or batches with uniformly short tails.

***The total number of findings exceeds the number of slaughtered batches, as multiple findings could be recorded for the same batch

**Table S4**. Post-mortem (PM) findings.

**Table S5.** The main causes of post-mortem (PM) condemnation in pigs in 15 Norwegian abattoirs, 2021-2024. *Overall frequencies were calculated as the number of carcasses with each finding divided by the total number of slaughtered pigs (n= 3 787 113) and expressed per 10 000 pigs. **Columns for total and partial condemnations show the number and percentage of carcasses totally or partially condemned for each finding.

**Table S6.**  Changes in variance components and VPC ratios across mixed models excluding the most influential abattoirs. The table presents the changes in residual variance (Δσ²) at the farm and abattoir level ($\Delta\sigma_{\mathrm{abattoirs}}^{2}, \Delta\sigma_{\mathrm{farm}}^{2}$), and the corresponding VPC ratios for the mixed models excluding the most influential abattoirs. Δσ² are the changes in variance components compared to the models with only random effects. Abattoir number reflects the numbering used in Figure 1. *Excluding abattoir 8, **excluding abattoir 3

**Table S7*.***  Changes in variance components and VPC ratios across mixed models with season as fixed effect. The table presents the changes in residual variance (Δσ²) at the farm and abattoir level ($\Delta\sigma_{\mathrm{abattoirs}}^{2}, \Delta\sigma_{\mathrm{farm}}^{2}$), and the corresponding VPC ratios for the mixed models including season as fixed effect for each EDR and PM finding. Δσ² represents the change in variance compared to the models with only random effects.
